# LINC00667/miR-449b-5p/YY1 axis promotes cell proliferation and migration in colorectal cancer

**DOI:** 10.1186/s12935-020-01377-7

**Published:** 2020-07-17

**Authors:** Juan Yu, Furang Wang, Jun Zhang, Jing Li, Xiaoguang Chen, Guangsen Han

**Affiliations:** 1 Endoscopic Diagnosis and Treatment Center, Affiliated Cancer Hospital of Zhengzhou University, No. 127, Dongming Road, Zhengzhou, 450003 Henan China; 2 General Surgery Department, Affiliated Cancer Hospital of Zhengzhou University, No. 127, Dongming Road, Zhengzhou, 450003 Henan China

**Keywords:** Colorectal cancer (RC), LINC00667, miR-449-45p, YY1

## Abstract

**Background:**

Long non-coding RNAs (lncRNAs) have been defined as vital regulators in the progression of human cancers, including colorectal cancer (CRC). Long intergenic non-protein coding RNA 667 (LINC00667) is a tumor promoter in several cancer types, while its role in CRC remains to be unmasked. This study focused on exploring the potential function and regulatory mechanism of LINC00667 in CRC.

**Methods:**

qRT-PCR analysis was applied to detect the expression of LINC00667 in CRC cells. Loss-of function assays revealed the role of LINC00667 silencing in regulating CRC cell proliferation, apoptosis and migration. In vivo study demonstrated the effect of LINC00667 silencing on CRC cell growth. Mechanism experiments were conducted to determine the upstream or the downstream molecular mechanism of LINC00667 in CRC cells.

**Results:**

LINC00667 was expressed at high level in CRC cells. LINC00667 knockdown significantly inhibited CRC cell growth and migration. YY1 transcription factor induced the upregulation of LINC00667 in CRC cells by transcriptionally activating LINC00667. In addition, miR-449b-5p could interact with LINC00667 in CRC cells. Intriguingly, miR-449b-5p directly targeted to YY1, thus inhibiting YY1 expression. YY1 recovered the CRC cell functions impaired by LINC00667 silencing.

**Conclusions:**

LINC00667 is transcriptionally activated by YY1 and promotes cell proliferation and migration in CRC by sponging miR-449b-5p to upregulate YY1.

## Background

Colorectal cancer (CRC) is recognized as a common malignancy in digestive system, with the fourth cancer-related mortality and increasing morbidity in the globe [1, 2]. Although advances have been made in the diagnosis and treatment of CRC patients, the 5-year survival rate is still only 10–15% [3]. Up to now, the underlying mechanisms associated with the initiation and development of tumors remain to be further understood.

Long noncoding RNA (lncRNA) is a class of non-protein coding transcripts that are longer than 200 nucleotides (nt) [4]. Previous studies have demonstrated that dysregulated lncRNAs have crucial roles in various biological processes, such as cell differentiation, proliferation, apoptosis and migration [5–7]. Evidence has been provided to show the functions of lncRNAs in tumorigenesis and malignant development of human cancers. For instances, lncRNA MIR205HG functions as a miR-122-5p sponge to accelerate tumor growth and progression of cervical cancer [8]. LncRNA FAM83H-AS1 is related with cell proliferation, invasion and migration in bladder cancer [9]. LncRNA GASL1 restrains cell proliferation by inhibiting E2F1 activity [10]. Long intergenic non-protein coding RNA 667 (LINC00667) is a new-founded lncRNA that has been demonstrated to be oncogenic in glioma [11] and non-small cell lung cancer [12]. Nevertheless, it is unknown whether LINC00667 functions in CRC.

Mounting evidence has revealed that lncRNAs can be activated by their upstream transcription regulators [13–15]. In our current study, the upstream molecular mechanism of LINC00667 was also explored. Mechanistically, lncRNAs can exert functions in human cancers by sponging certain miRNAs to release the downstream mRNAs [16–18]. It is unclear whether LINC00667 exert functions in CRC in the same way.

To summarize, this study focused on exploring the functions of LINC00667 in CRC and its upstream or downstream molecular mechanism.

## Methods

### Patient samples

The procedures of clinical study had obtained the approval of the Ethic committee of Affiliated Cancer Hospital of Zhengzhou University. Before this study, the written informed consent had obtained from three participants who were diagnosed with CRC. Three pairs of fresh CRC tissues and adjacent normal tissues were collected for surgical resection. None of participants received any chemotherapy or radiotherapy prior to surgery. Samples were snap frozen in liquid nitrogen and stored at − 80 °C.

### Cell culture

The human colon epithelium cell line (FHC) and human CRC cell lines (HT29, SW620, LoVo and HCT116) were obtained from ATCC (Manassas, VA, USA). All cell lines were maintained in RPMI 1640 (Gibco Laboratories, Grand Island, NY, USA) containing 10% fetal bovine serum (FBS; Gibco) and antibiotics (100 U/mL penicillin and 100 μg/mL streptomycin; Gibco) under humidified condition with 5% CO_2_ at 37 °C.

### Quantitative real time polymerase chain reaction (qRT-PCR)

Total RNA of cultured cells was extracted using TRIzol (Invitrogen, Carlsbad, CA, USA) to detect the RNA expression. 1 μg/reaction RNA was reverse transcribed into cDNA (1 μl per tube) by using Biosystems High-Capacity cDNA Reverse Transcription Kit (Invitrogen). 20 μl RNA was used in the whole reaction system. RNA quality was measured by molecular devices SPECTRA MAX190. The spectrophotometer was reset using TE solution. RNA solution was diluted with TE (1:100) and the absorbance was measured at the wavelength of 260 nm and 280 nm. The thermal cycling profile was as follows: denaturation for 30 s at 95 °C, annealing for 45 s at 56 °C, and extension for 45 s at 72 °C. Each PCR reaction was carried out for 35 cycles using the ABI 7500 Real-Time PCR system (Applied Biosystems, Foster City, CA, USA). For the detection of miRNA expression, the Applied Biosystems TaqMan MicroRNA Assay protocol (Applied Biosystems, Carlsbad, CA, USA) was applied to conduct reverse transcription. The SYBR PrimeScriptTM miRNA RT-PCR Kit (TaKaRa Biotech) was used to examine miRNA expression. The RNA expression levels were calculated by the 2^−ΔΔCt^ method. The requirements for use of 2^−ΔΔCt^ method have been fulfilled. Ct value for GAPDH and LINC00667 was listed in Supplementary Table 1 (Additional file 1: Table S1). GAPDH or U6 was used as an endogenous control. qRT-PCR analysis was performed in accordance with previous guidance [19]. Primers’ sequences were listed in Table 1. Each sample was analyzed in triplicate. All experimental procedures were repeated for three times.

**Table 1:** PCR primers

LINC00667	F TGTGCGAGAAAGCCTACCTG
LINC00667	R GCCTGCATCAAAAAGTCGGG
miR-449b-5p	F GCCGAGAGGCAGTGTATTGTTA
miR-449b-5p	R AGGCAGTGTATTGTTAGCTGGC
YY1	F AAGTGCATTCCACCCGAACT
YY1	R AAGGGCCTGCACTTAAACCA
GAPDH	F GGAGCGAGATCCCTCCAAAAT
GAPDH	R GGCTGTTGTCATACTTCTCATGG
U6	F CTCGCTTCGGCAGCACA
U6	R AACGCTTCACGAATTTGCGT

### Cell transfection

For the stable knockdown of LINC00667 or YY1 [20], short hairpin RNAs (shRNAs) targeting LINC00667 (shLINC00667#1/2/3) or YY1 (shYY1) and the negative control shRNA (shNC) were purchased from GeneCopoeia, Inc. (Rockville, MD, USA). MiR-449b-5p mimics, miR-449b-5p inhibitor, and negative control (NC mimics, NC inhibitor) were all purchased from Shanghai GenePharma Inc. (Shanghai, China), along with the YY1-overexpression vector and control (pcDNA3.1). The above plasmids were transfected into HCT116 and LoVo cells using Lipofectamine^®^ 2000 transfection reagent (Invitrogen) according to the manufacturer’s protocol for 48 h. All experimental procedures were repeated for three times. The shRNA sequences for LINC00667 were listed as follows:

shNC: 5′-CCGGCAGATTAGTCTCAACTTGACTCTCGAG AGTCAAGTTGAGACTAATCTG TTTTTG-3′;

shLINC00667#1: 5′-CCGGAAGTTTGACCCTGATTCTCAACTCGAG TTGAGAATCAGGGTCAAAC TTTTTG-3′;

shLINC00667#2: 5′-CCGGTACATGTTTGGTAGAGAACTACTCGAG TAGTTCTCTACCAAACATGTA TTTTTG-3′;

shLINC00667#3: 5′-CCGGTAAACAATAGTGTAGTAACTACTCGAGTAGTTACTACACTATTGTTTA TTTTTG-3′.

### Bioinformatics analysis

Based on the protocols [21, 22], the potential transcription factors of LINC00667 were downloaded from PROMO tool of ALGGEN dataset (http://alggen.lsi.upc.es/) and subjected to qRT-PCR analysis in one normal colon epithelial cell and four CRC cells. Heatmap represented the differential genes using Cluster 3.0 software (http://hemi.biocuckoo.org).

### CCK-8 assay

As previously described [23], cell viability of CRC cell lines was determined by Cell Counting Kit-8 (CCK-8) in a 96-well plate as per standard procedure. LoVo and HCT116 cells (1 × 10^3^ cells/well) treated with indicated transfection plasmids were incubated to 80%–90% confluence. Cells were pre-incubated at 37 °C in 5% CO_2_ atmosphere, and CCK-8 reagent was added into each well (10 μl) at five different time points (0, 24, 48, 72, 96 h) and cultured at 37 °C in 5% CO_2_ for 4 h. The absorbance was measured by a microplate reader (Thermo Fisher Scientific, Waltham, MA, USA) at 450 nm to determine cell viability. All experimental procedures were repeated for three times.

### 5-Ethynyl-2′-Deoxy-uridine (EdU) assay

Based on the instruction of EdU labeling/detection kit (RiboBio, Guangzhou, China), the EdU incorporation assay was performed to evaluate cell proliferation [24]. Cells were cultured with 50 μM of EdU diluent at 37 °C with 5% CO_2_ for 2 h. And then 4% paraformaldehyde was used to fix cells for 30 min. After cells were washed with PBS, cells were then stained with Apollo 567 working solution for 30 min. Cells were observed and captured using fluorescent microscopy (Thermo Fisher Scientific). All experimental procedures were repeated for three times.

### Caspase-3 activity assay

As previously described [25], the apoptosis of transfected LoVo or HCT116 cells was evaluated by using the Caspase-3 Activity Assay Kit (Solarbio, Beijing, China). In short, extracted proteins from the transfected cells were into 96-well plates containing the reaction buffer and caspase-3 substrate. Caspase-3 activity was measured by a microplate reader (Leica) at 405 nm. All experimental procedures were repeated for three times.

### Transwell migration assay

As previously described, transwell assays were performed to assess cell migration [26]. After 48 h of transfection, 2 × 10^4^ cells were added to the upper chamber containing 100 μl serum-free medium and then 10% FBS (Gibco) was added in the lower chamber. After 24 of incubation, the migrated cells through the pores were fixed and stained with crystal violet. Cells were observed and photographed under the microscope (Leica, Shanghai, China). All experimental procedures were repeated for three times.

### In vivo model

Six weeks old BALB/c male mice were purchased from Guangdong Medical Laboratory Animal Center (Guangdong, China). In vivo experiments were approved by the Institutional Animal Care and Use Committees of Affiliated Cancer Hospital of Zhengzhou University. Briefly, after stable transfection with shNC or shLINC00667#2, 2 × 10^6^ HCT116 cells were subcutaneously injected into the flanks of mice. N = 3 in each group. Tumors were observed every 4 days. Tumor volume was calculated as (Length  ×  Width^2^/2). Twenty-eight days later, the mice were sacrificed, tumor weight was measured.

### RNA fluorescent in situ hybridization (RNA FISH)

The cellular distribution of LINC00667 was detected by performing FISH assay [27]. Cells were plated in 24 well plates at a density of 6 × 10^4^ cells. When the confluence reached to 60%–70%, FISH assay was performed by use of Ribo™ Fluorescent in Situ Hybridization Kit (C10910, Ribobio). Cells were fixed with 4% for 30 min and treated with 0.5% TritonX-100 at 4 °C for 5 min. The nuclei were stained with DAPI and washed thrice with PBS. The sequence for LINC00667 probe used in FISH assay was presented in Table 2. The designed and synthesized LINC00667 probes were labeled with fluorescent dye. Fluorescence signals were performed with a confocal laser-scanning microscope (Leica). All experimental procedures were repeated for three times. All experimental procedures were repeated for three times.

**Table 2:** Sequence for LINC00667 probe used in FISH assay

Gcuugacugucuucaacaaaccaaugccacauuuaaaauguuuaaacauuaaccuucccagucccagguaugaugcuuuguguuacuugugguaucuaucucucucucucucugucaaucacacacacacacacacagccuacagguagguaguccagggcugguauugucauuccacaucuagaauccagcuuucagguccacccuuccuggggagugacucucaucuucauaaucuaaaauggcugcuaggguguuagccaucacauuugcauuccaggcagaaaaaugaagaaaggaaaaagaaggcaaagggcaauguucuuacugucuuuaauaguuuccuaacacugccacagaagauaucugcuuauaucucacugucuaaaguucagucacauggccacaaucucaaggaaggcugggguacuuuaaucuuuauugagggcagucaugugcccagcuaaaaccagggguucuauuacuaagaaagauacagcugccccccaccccaaaacaauucaauaaaaauacaaauaaacaauaguguaguaacuauuuucauagcauuuacacauucauuauuauaaguaauguagagaugauuuaaaguauauggaagaugugcaaagguuauaugcaaauacu guaauauuuuauauaaaugacuugagcaccugcagauuuugguaucccugagaguuccuggaaccaauccccuucagauaccaacgaauaacuguacauguuugguagagaacuaguugucucuaccuagucuccauucuggucacuucuuuaguuuccuaauuucagaguaaggccagucuccuucugugaugguuaauuuugugucaacuugagugaaccaagggaugcccagauaccugguaaaacauuauuuccacguguguuggugaggguguuucuggaagucauugacauuucuacugguagacugaguaaagaagauccacccucacuaauguggaugggcauca	

### Subcellular fractionation

Subcellular fractionation was conducted as previously described to identify the cellular location of LINC00667 [28]. Using the nuclear or cytoplasmic Isolation Kit (Biovision, San Francisco, CA, USA), the cytosolic and nuclear fractions of LoVo or HCT116 cells were separately isolated. Then, the expression ratio of RNA molecules in the cytoplasm and nucleus were measured using qRT-PCR, normalizing to U6 (nuclei control) and GAPDH (cytoplasm control). All experimental procedures were repeated for three times.

### RIP assay

For RIP assay [29], LoVo and HCT116 cells were collected and lysed in RIP lysis buffer. Next, magnetic beads were incubated with anti-Ago2 (abcam, Cambridge, USA; ab32381) or anti-IgG control (abcam, ab172730) overnight at 4 °C. The precipitated RNA was isolated, purified and reverse transcribed. The relative enrichment of LINC00667, YY1 and miR-449b-5p were analyzed by qRT-PCR. All experimental procedures were repeated for three times.

### Chromatin immunoprecipitation (ChIP) assay

ChIP assay was carried out as previously described [30]. Cells were treated with 1% methanol, incubated at room temperature for 10 min and then lysed. The protein was extracted and sonicated to obtain the soluble chromatin. The immunoprecipitated RNA was collected and incubated overnight with anti-YY1 or control IgG. The recovered DNA was purified from the immune complex and then was subjected to qRT-PCR. All experimental procedures were repeated for three times.

### Luciferase reporter assays

Partial wild-type or mutant DNA sequences of miR-449b-5p binding sites in LINC00667 and YY1 3′ UTR were amplified using PCR. Then, they were sub-cloned respectively into pGL3 vector (Promega, Madison, WI, USA) to produce LINC00667-WT, LINC00667-MUT, YY1-WT, and YY1-MUT reporter plasmids. Afterwards, LoVo or HCT116 cells were co-transfected with transfection plasmids and constructed reporter plasmids using Lipofectamine^®^ 2000 (Invitrogen). Cells were collected at 48 h transfection. Under different treatment conditions, the luciferase activity in cells was determined using the Dual-Luciferase^®^ Reporter Assay kit (Promega) in line with manufacturer’s instructions [31]. All experimental procedures were repeated for three times.

For the investigation of the impact of transcription factors on LINC00667 promoter, luciferase reporter assay was conducted as previously described [32]. Briefly, LINC00667 promoter region was sub-cloned into pGL3 vector to generate LINC00667 promoter reporter plasmids. And then, the plasmids were co-transfected with different shRNAs targeting PBF, Sp1, E2F, YY1 and WT1. In return, YY1 promoter region was cloned into pGL3 vector to generate YY1 promoter reporter plasmids with the co-treatment of shNC or shLINC00667#2. As for the binding affinities of predicted miRNAs with YY1, YY1 3′ UTR containing the possible binding sequences for miRNAs was established into pGL3 vectors was co-transfected with overexpressed miRNAs into HCT116 and LoVo cells. All experimental procedures were repeated for three times.

### Western blot

Western blot analysis was made to assess proteins in transfected cells in accordance with a previous study [33]. RIPA lysis buffer with a protease inhibitor cocktail was prepared to lyse cells. Next, total protein was separated by SDS PAGE and transferred onto PVDF (polyvinylidene difluoride) membranes. After being blocked with 5% non-fat milk, the membrane was incubated with primary antibodies: anti-Bax (abcam, ab32503), anti-Bcl-2 (abcam, ab185002), anti-YY1 (abcam, ab109237), and anti-GAPDH (abcam, ab8245) at 4 °C overnight. After incubation with specific antibodies, the blots were incubated with the secondary antibody and visualized with enhanced chemiluminescence. Then, GAPDH was used as an internal control. All experimental procedures were repeated for three times.

### Statistical analysis

Statistics of three repeated experiments were analyzed using GraphPad Prism 6 software (GraphPad Software, Inc., La Jolla, CA, USA) and compared using Student’s t-test or one-way analysis of variance (ANOVA). Data are presented as the mean ± SD. Differences were deemed as statistically significant when P < 0.05.

## Results

### Silencing of LINC00667 suppresses cell proliferation and migration in CRC

**Fig. 1:**
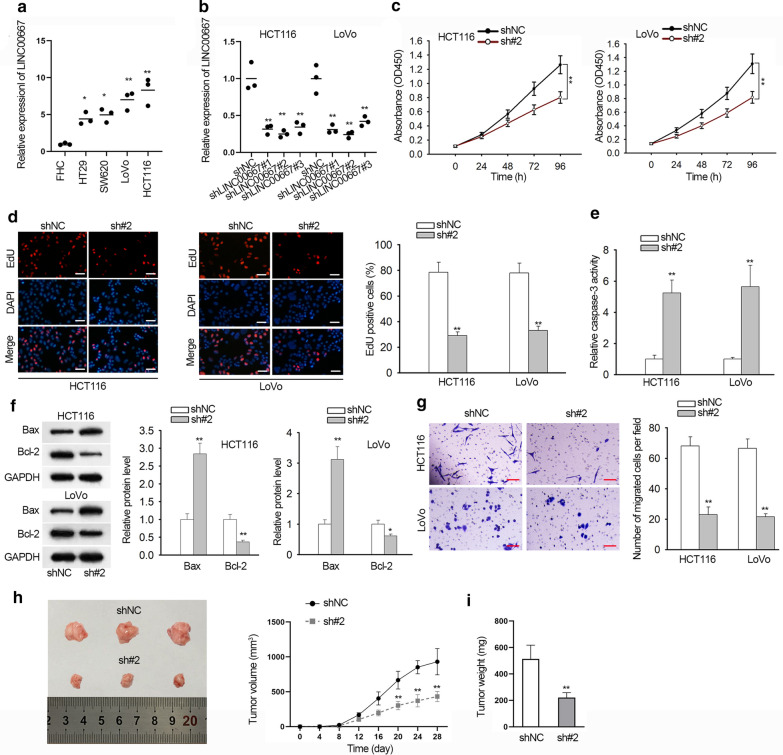
LINC00667 acts as an oncogene to promote CRC cell proliferation and migration**. a** The expression of LINC00667 in the human colon epithelium cell line (FHC) and four human CRC cell lines (HT29, SW620, LoVo and HCT116). Results were obtained using qRT-PCR (n = 3 in each group). **b** The interfering efficiency of shRNAs specific to LINC00667 was tested by qRT-PCR at 48 h after transfection (n = 3 in each group). **c** The viability of CRC cells transfected with shNC or shLINC00667#2 for 48 h was measured by CCK-8 assay in several different time points (0 h, 24 h, 48 h, 72 h, 96 h). N = 3 in each group. **d** The proliferation of LINC00667-silenced cells was detected by EdU assay after treated with shRNAs for 48 h. Red: EdU, blue: DAPI. N = 3 in each group. Scale bar = 200 μm. **e** Caspase-3 activity was assessed by measuring the wavelength at 405 nm. N = 3 in each group. **f** The levels of apoptosis-related proteins (Bax and Bcl-2) were measured by western blot analysis in HCT116 and LoVo cells after transfected with shNC or shLINC00667#2. N = 3 in each group. **g** Transwell assay measured the migratory ability of LINC00667-silenced cells. N = 3 in each group. Scale bar = 200 μm. **h** Tumors derived from HCT116 cells stably transfected with shNC or shLINC00667#2 were shown. Tumor volume was measured every four days. n = 3 in each group. **i** Tumor weight in two different groups were shown. n = 3 in each group. ^*^P < 0.05, ^**^P < 0.01 compared to control group

LINC00667 (ENST00000582008.5) is located at chr18:5,238,099-5246,507, which is consisted with 3 exons and 2 introns. At first, we measured the level of LINC00667 in CRC cells and normal colon epithelial cell. The high level of LINC00667 was observed in CRC cells (Fig. 1a). Considering the highest level of LINC00667 in LoVo and HCT116 cells, we conducted loss-of function assays in these two cells. In subsequence, LINC00667 was silenced by shRNAs targeting LINC00667 (shLINC00667#1, shLINC00667#2 and shLINC00667#3) in LoVo and HCT116 cells with non-targeting shRNA as negative control (shNC). The result of qRT-PCR analysis revealed that the level of LINC00667 was obviously decreased by three specific shRNAs, especially by shLINC00667#2 (Fig. 1b). Therefore, shLINC00667#2 was chosen for functional experiments. CCK-8 assay showed that cell viability was repressed with the downregulation of LINC00667 in LoVo and HCT116 cells (Fig. 1c). EdU assay showed that EdU-positive cells were decreased in LINC00667ilenced CRC cells compared to control cells (Fig. 1d). Through the detection of caspase-3 activity, the activity of caspase-3 was found to be enhanced in cells transfected with shLINC00667#2 compared to those transfected with shNC (Fig. 1e). Western blot analysis further validated that the apoptosis was promoted by the silencing of LINC00667 in accordance with the increased level of Bax and the decreased level of Bcl-2 (Fig. 1f). Besides, transwell assay demonstrated that the migratory ability of LoVo and HCT116 cells was impaired by the silencing of LINC00667 (Fig. 1g). At last, In vivo experiment further demonstrated that silencing of LINC00667 suppressed CRC cell growth in vivo (Fig. 1h, i). Taken together, LINC00667 inhibition suppresses CRC cell growth and migration.

### YY1 regulates LINC00667 expression by acting as a transcription activator

**Fig. 2:**
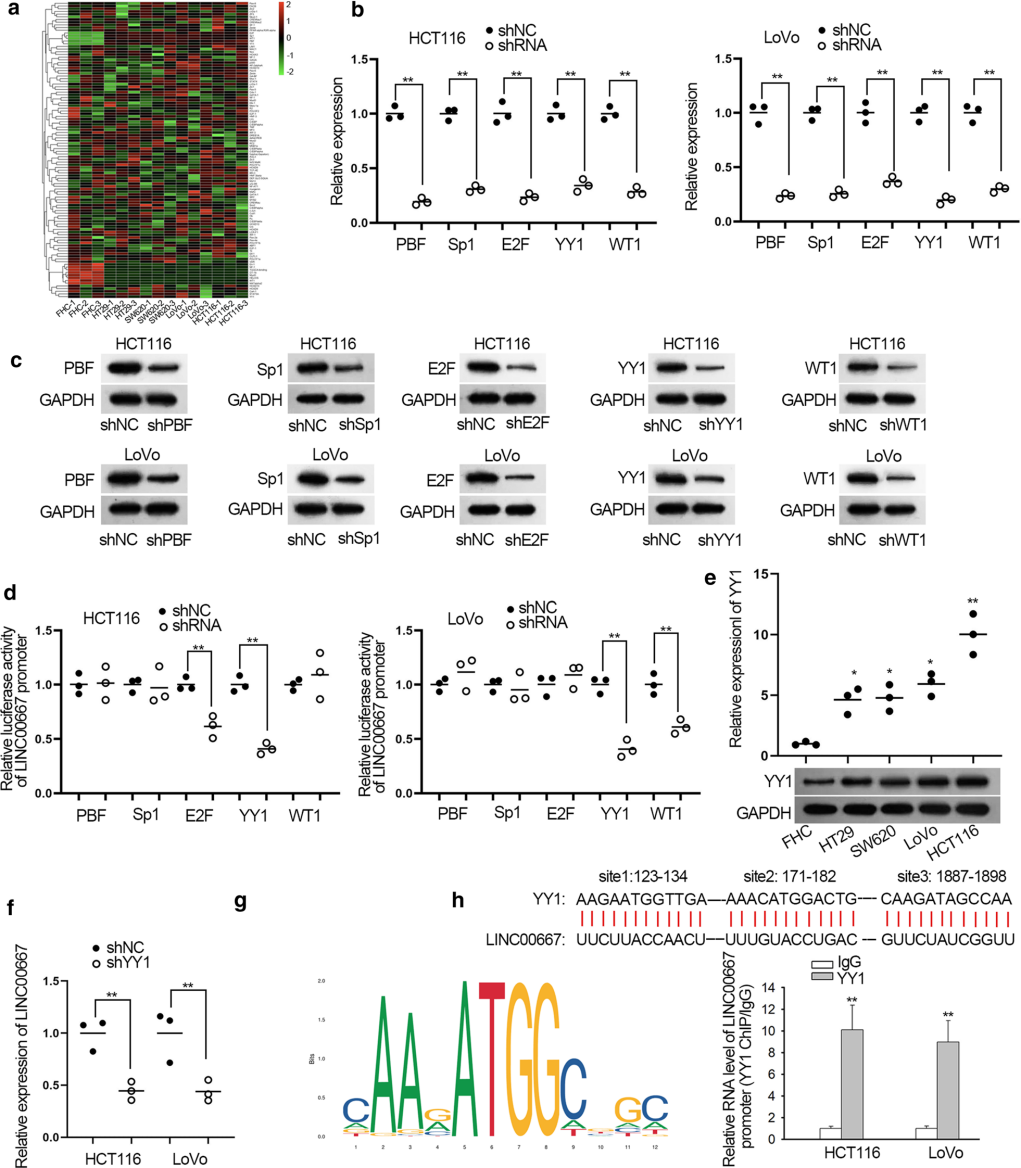
YY1 transcriptionally activates LINC00667 in CRC cells. **a** All potential transcription factors in the upstream of LINC00667 were subjected to qRT-PCR analysis in CRC cells with the normal colon epithelial cells as the negative control. Results were shown as a heatmap. **b**, **c** The mRNA and protein levels of five candidate transcription factors were detected in cells transfected with specific shRNAs. Results were examined through qRT-PCR and western blot after 48 h’ transfection. N = 3 in each group. **d** Luciferase reporter assays were performed to measure the luciferase activity of reporter vector containing the whole sequence of LINC00667 promoter after co-transfected with specific shRNAs for 48 h. N = 3 in each group. **e** The levels of YY1 mRNA and protein in CRC cells and normal FHC cell were measured by qRT-PCR and western blot, respectively. N = 3 in each group. **f** LINC00667 expression was tested via qRT-PCR after silencing of YY1. N = 3 in each group. **g** DNA motif of YY1 transcription factor downloaded from JASPAR was shown. **h** Three binding sequences of YY1 in LINC00667 promoter were exhibited. The role of YY1 as a transcription factor of LINC00667 was verified by ChIP assay in HCT116 and LoVo cells. N = 3 in each group. ^*^P < 0.05, ^**^P < 0.01 compared to control group

Transcription factors are known as regulators of genes in nucleus [34]. Here, we predicted the potential upstream transcription factors of LINC00667 by using the online tool ALGGEN. The levels of these genes were detected by qRT-PCR analysis in CRC cells and results were presented in Fig. 2a. Top five overexpressed transcription factors in CRC cells were chosen for subsequent analyses. We silenced these genes via specific shRNAs and the decreased levels were determined separately by qRT-PCR and western blot (Fig. 2b, c). In luciferase reporter assay, only YY1 down-regulation restrained the activity of LINC00667 promoter in both CRC cells (Fig. 2d). Thereafter, YY1 was chosen for follow-up experiments. Through qRT-PCR and western blot, the levels of YY1 mRNA and protein were higher in CRC cells, especially in HCT116 and LoVo cells (Fig. 2e). Meanwhile, the protein level of YY1 was remarkably higher in CRC tissues compared to adjacent normal tissues (Additional file 2: Figure S1A). The expression of LINC00667 was also decreased with the knockdown of YY1 (Fig. 2f). Next, we analyzed the interaction between YY1 and LINC00667 promoter. The DNA motif of STAT3 was obtained from JASPAR (Fig. 2g) and the binding sequences between YY1 and LINC00667 promoter were shown (Fig. 2h, upper panel). ChIP assay confirmed the affinity of YY1 to LINC00667 promoter (Fig. 2h). These data showed that YY1 transcriptionally activates LINC00667 by binding to LINC00667 promoter.

### LINC00667 acts as a sponge for miR-449b-5p in CRC cells

**Fig. 3:**
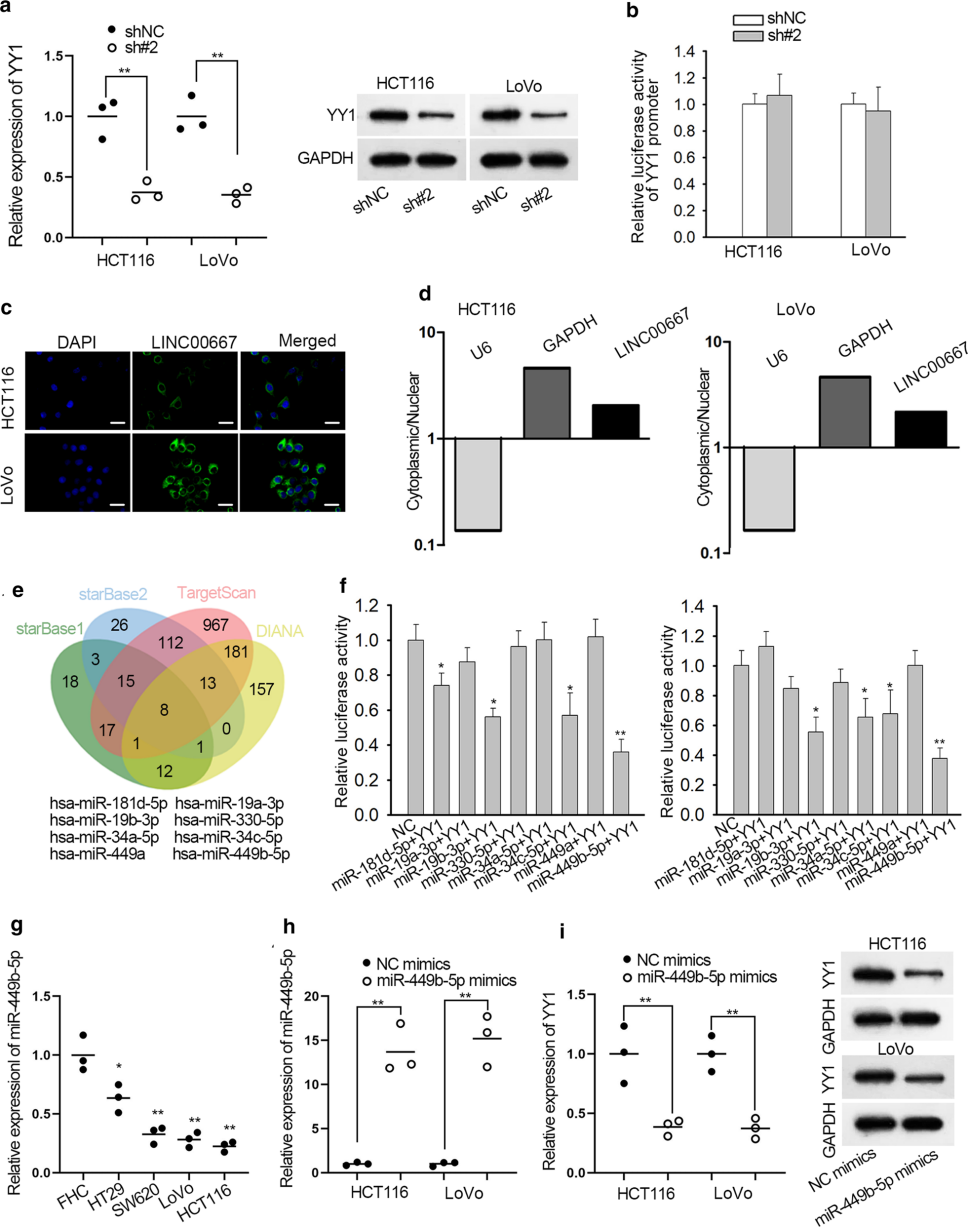
MiR-449b-5p potentially interacts with both LINC00667 and YY1. **a** qRT-PCR analysis of YY1 expression after transfected with LINC0067-specific shRNAs for 48 h. N = 3 in each group. **b** Promoter luciferase reporter assay was conducted to measure the luciferase activity of YY1 promoter vector in LINC00667-silenced cells. N = 3 in each group. **c** FISH assay revealed the localization of LINC00667 in CRC cells. Blue: DAPI, green: LINC00667. N = 3 in each group. Scale bar = 100 μm. **d** The ratio of cytoplasm to nucleus was detected by subcellular fractionation assay, Results were analyzed by qRT-PCR. N = 3 in each group. **e** Using starBase, DIANA and TargetScan tools, eight miRNAs were predicted to potentially bind to both LINC00667 and YY1. **f** The binding affinity of eight miRNAs with YY1 was explored by luciferase reporter assays. n = 3 in each group. **g** The expression of miR-449b-5p in four CRC cells and one colon epithelial cell line (FHC) was measured by qRT-PCR. N = 3 in each group. **h** MiR-449b-5p expression in two CRC cells transfected with miR-449b-5p mimics or NC mimics for 48 h. N = 3 in each group. **i** The levels of YY1 mRNA and protein were measured in HCT116 and LoVo cells transfected with miR-449b-5p mimics or NC mimics for 48 h. N = 3 in each group. ^*^P < 0.05, ^**^P < 0.01 compared to control group

Above findings have shown that LINC00667 is a target of YY1. Since previous studies have shown that YY1 can be regulated by lncRNAs. In this study, we explored whether LINC00667 could regulate YY1 in turn. Accordingly, the level of YY1 mRNA and protein were measured in two CRC cells transfected with shNC or shLINC00667#2. Intriguingly, both levels of YY1 were reduced after silencing of LINC00667 (Fig. 3a), indicating the regulatory effect of LINC00667 on YY1. Luciferase reporter assay revealed that LINC00667 has no significant effect on the luciferase activity of reporter vector containing YY1 promoter (Fig. 3b), excluding the transcriptional regulation of LINC00667 on YY1. According to the results of FISH and subcellular fractionation assays, LINC00667 was mainly located in the cytoplasm of HCT116 and LoVo cells (Fig. 3c, d). It have been reported that cytoplasmic lncRNAs can exert functions in human cancers through sponging miRNAs to upregulate mRNAs [35, 36]. Here, we speculated that LINC00667 modulated YY1 in CRC in the same way. Then, we predicted the miRNAs binging with both LINC00667 and YY1 from starBase v3.0, DIANA and TargetScan. The predicted results were shown as a Venn diagram (Fig. 3e), which presented that there were eight shared miRNAs of LINC00667 and YY1. Among these miRNAs, merely miR-449b-5p showed the highest binding ability with YY1 in both HCT116 and LoVo cells (Fig. 3f). Besides, miR-449b-5p expression was down-regulated in CRC cells (Fig. 3g). Both mRNA and protein levels of YY1 were lowered when miR-449b-5p was overexpressed (Fig. 3h, i). These findings revealed that miR-449b-5p may be a media for the regulatory mechanism between LINC00667 and YY1.

### LINC00667 acts as miR-449b-5p decoy to mediate YY1

**Fig. 4:**
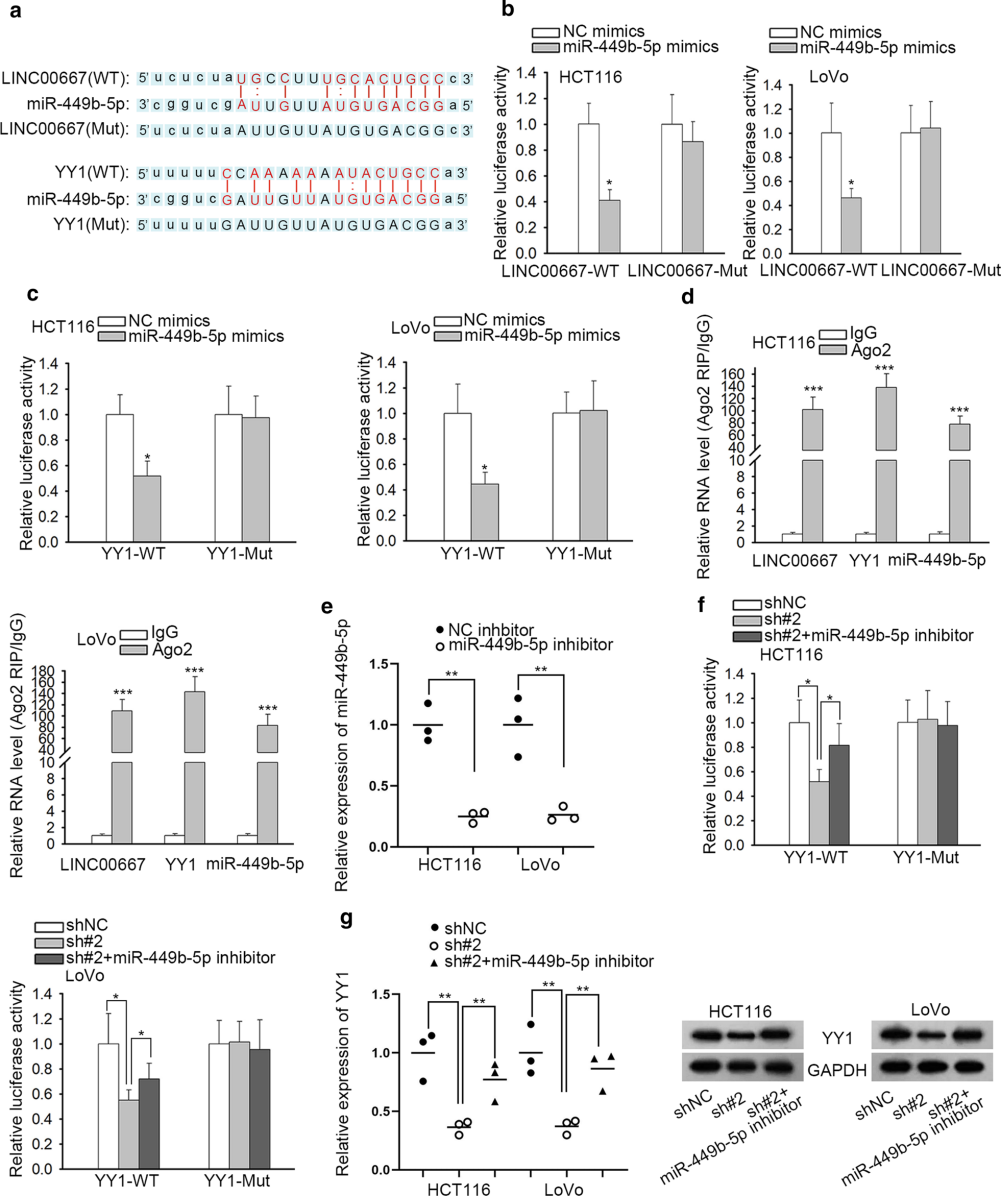
LINC00667 enhances YY1 expression by sequestering miR-449b-5p in CRC cells. **a** The putative binding sites of miR-449b-5p in LINC00667 or YY1 3′-UTR. **b** Luciferase reporter assay was conducted in two CRC cells to determine the interaction between miR-449b-5p and LINC00667. N = 3 in each group. **c** The luciferase activity of YY1-WT or YY1-Mut vector was measured after co-transfected with NC mimics or miR-449b-5p mimics. N = 3 in each group. **d** Ago2-RIP assay was performed in two CRC cells to identify the enrichment of LINC00667, miR-449b-5p and YY1 in the immunoprecipitates conjugated to Ago2 antibody. N = 3 in each group. **e** MiR-449b-5p expression was decreased after transfected with miR-449b-5p inhibitor for 48 h. NC inhibitor was used as negative control. N = 3 in each group. **f** The luciferase intensity of YY1-WT or YY1-Mut reporter plasmid was detected in cells co-transfected with shNC, shLINC00667#2 or shLINC00667#2 + miR-449b-5p inhibitor. N = 3 in each group. **g** The mRNA and protein levels of YY1 were measured in two CRC cells transfected with shNC, shLINC00667#2 or sh-LINC00667#2 + miR-449b-5p inhibitor. N = 3 in each group. ^*^P < 0.05, ^***^P < 0.001 compared to the control group

Subsequently, we searched on starBase v3.0 and obtained the binding sequences of LINC00667 or YY1 for miR-449b-5p, and the mutated binding sequences were also gained (Fig. 4a). The interaction between miR-449b-5p and LINC00667 or YY1 was determined through luciferase reporter assays. As a result, the luciferase activity of LINC00667-WT or YY1-WT was hampered by miR-449b-5p overexpression (Fig. 4b, c). According to the results of Ago2-RIP assay, we confirmed the co-existence of LINC00667, YY1 and 305 miR-449b-5p in RISC (Fig. 4d). In order to probe the co-influences of LINC00667 and miR-449b-5p on YY1, we firstly decreased miR-449b-5p expression by transfection of miR-449b-5p inhibitor (Fig. 4e). In luciferase reporter assay, the luciferase activity of YY1-WT was initially repressed by LINC00667 silencing but recovered by miR-449b-5p inhibition (Fig. 4f). Further qRT-PCR and western blot analyses indicated that the LINC00667 repression-decreased levels of YY1 mRNA and protein were recovered by miR-449b-5p obstruction (Fig. 4g). All in all, LINC00667 upregulates YY1 expression by sponging miR-449b-5p.

### YY1 overexpression abolishes the inhibitory effects of LINC00667 knockdown on CRC cell growth and migration

**Fig. 5:**
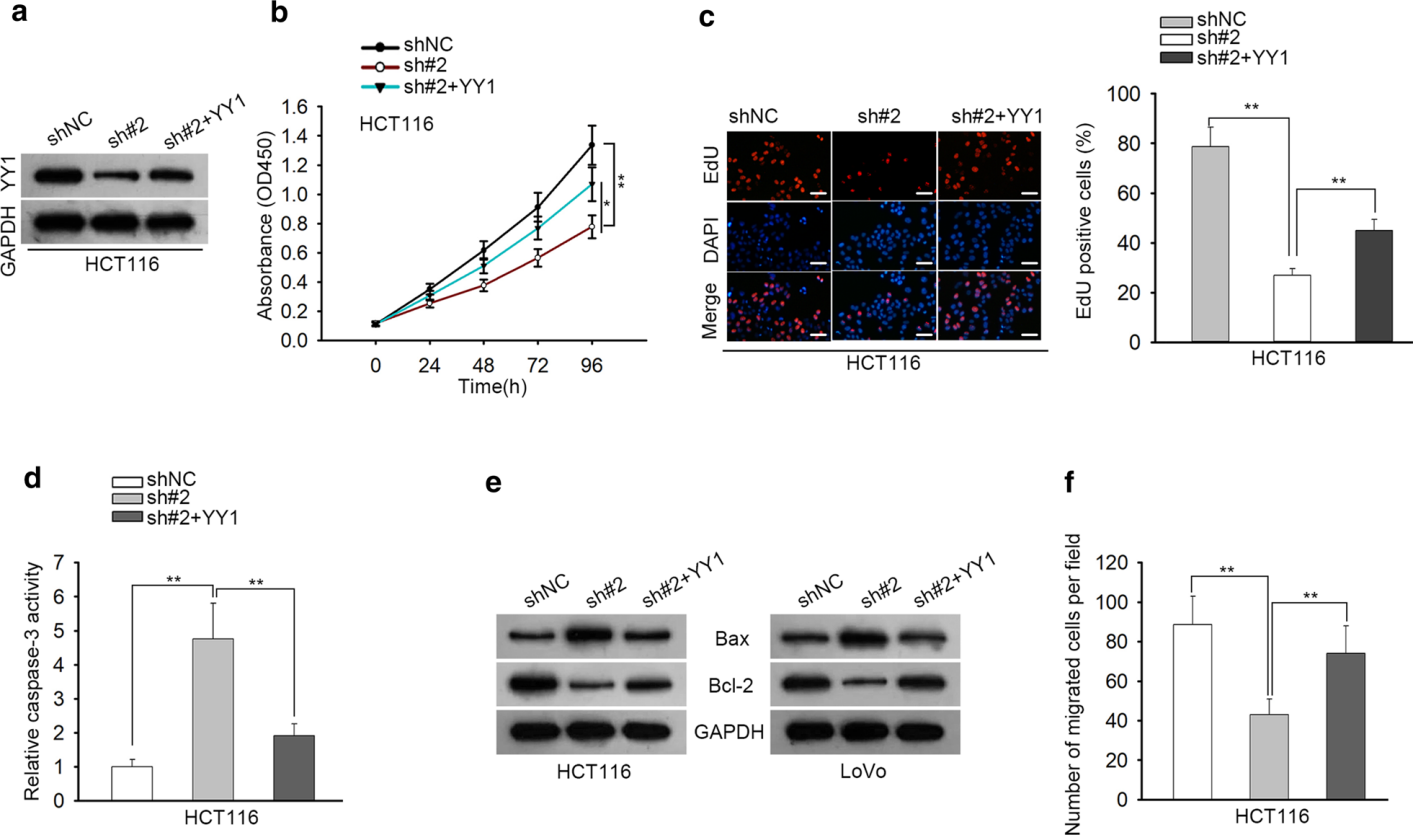
LINC00667/miR-449b-5p/YY1 axis modulates CRC cell proliferation and migration. **a** The levels of YY1 protein were measured in HCT116 cells transfected with shNC, shLINC00667#2 or sh-LINC00667#2 + pcDNA3.1/YY1. N = 3 in each group. **b**–**f** HCT116 transfected with shNC, shLINC00667#2 or sh-LINC00667#2 + pcDNA3.1/YY1 were subjected to CCK-8 assay, EdU assay (Scale bar = 200 μm), Caspase-3 activity test and western blot analysis. N = 3 in each group. ^*^P < 0.05, ^**^P < 0.01 compared to the control group

Rescue assays were carried out to validate the whole mechanism of LINC00667 in CRC. HCT116 cells were transfected with shNC, sh#2 and sh#2 + YY1 separately. The protein levels of YY1 reduced by LINC00667 silencing were rescued by YY1 overexpression (Fig. 5a). In CCK-8 and EdU assays, the repressive effect of LINC00667 silencing on cell proliferation was counteracted by YY1 up-regulation (Fig. 5b, c). Caspase-3 activity test presented that LINC00667 silencing induced cell apoptosis and this effect was suppressed when YY1 was overexpressed (Fig. 5d). Consistently, the increased level of Bax and the decreased level of Bcl-2 caused by LINC00667 knockdown were reversed by the overexpression of YY1 (Fig. 5e). Transwell assays found that cell migration was restrained after LINC00667 was silenced, which was neutralized after YY1 was upregulated (Fig. 5f). In summary, LINC00667 regulates the proliferation, apoptosis and migration capacities in CRC through upregulating YY1.

## Discussion

Long non-coding RNAs (lncRNAs) exert important functions in the biological processes of different cancers, including CRC. For instance, highly expressed lncRNA AFAP1-AS1 promotes colon cancer progression and indicates poor prognosis [37]. LncRNA plasmacytoma variant translocation 1 (PVT1) boosts colon cancer progression by sponging miR-26b [38]. LncRNA B3GALT5-AS1 restricts colon cancer liver metastasis by repression of miR-203 [39]. LncRNA OCC-1 hinders cell growth via destabilizing HuR protein in colorectal cancer [40]. LINC00667 has been demonstrated to promote the vasculogenic mimicry of glioma cells [11]. The current study revealed the upregulation of LINC00667 in CRC cells for the first time. Importantly, silencing of LINC00667 suppressed CRC cell growth both in vitro and in vivo, indicating the oncogenic role of LINC00667 in CRC.

LncRNAs can be upregulated by their upstream transcriptional activators. For example, STAT3-induced the transcriptional activation of lncRNA lncRNA HAGLROS and strengthens the oncogenic potential of HAGLROS in gastric cancer [32]. LncRNA SPRY4-IT1 is activated by SP1 and contributes to the malignant progression of cholangiocarcinoma [14]. A series of assays validated that YY1 transcription factor (YY1) was screened to be the transcription factor most influencing LINC00667 expression. In previous studies, YY1 can promote the initiation and development of human carcinomas, including breast cancer [41], nasopharyngeal carcinoma [42], glioblastoma [43], thyroid cancer [44], non-small cell lung cancer [45] and so on. The modulation of YY1 on LINC00667 at transcriptional level was certified in our paper. Based on the cytoplasmic location of LINC00667, we then observed the post-transcriptional regulation of LINC00667 on YY1. Through mechanism investigation, miR-449b-5p with the highest binding ability for LINC00667, was selected for further exploration. The tumor suppressive role of miR-449b-5p has been probed in glioma, breast cancer and cervical cancer [46–48]. Here, we verified the interaction between LINC00667 and miR-449b-5p and explored the mechanism of LINC00667/miR-449b-5p/YY1 axis in CRC.

## Conclusion

In conclusion, LINC00667/miR-449b-5p/YY1 axis regulates the proliferation and migration of CRC cells. These data expose a potent and promising therapeutic target for the treatment of CRC patients.

## Supplementary information

**Additional file 1: Table S1.** Ct value for GAPDH and LINC00667.

**Additional file 2: Figure S1A.** The protein level of YY1 in three pairs of CRC tissues and adjacent normal tissues was measured by western blot analysis. N=3 in each group.

## Data Availability

Research Data are not shared.

## Abbreviations

lncRNAs: Long non-coding RNAs
ceRNA: Competing endogenous RNA
miRNAs: microRNAs
mRNA: Messenger RNA
COAD: Colon adenocarcinoma
LINC00667: Long intergenic non-protein coding RNA 667
YY1: YY1 transcription factor
RISC: RNA-induced silencing complex
ncRNAs: Non-coding RNAs
nt: Nucleotides
ATCC: American Type Culture Collection
RPMI: Roswell Park Memorial Institute
FBS: Fetal Bovine Serum
qRT-PCR: Quantitative real-time polymerase chain reaction
GAPDH: Glyceraldehyde-3-phosphate dehydrogenase
shRNAs: Short hairpin RNAS
CCK-8: Cell counting kit-8
EdU: 5-Ethynyl-2′-Deoxy-uridine
RNA FISH: RNA fluorescent in situ hybridization
RIP: RNA immunoprecipitation
ChIP: Chromatin Immunoprecipitation
UTR: Untranslated region
Wt: Wild-type
Mut: Mutant
PVDF: Polyvinylidene difluoride
SDS-PAGE: Sodium dodecyl sulfate–polyacrylamide gel electrophoresis
ANOVA: Analysis of variance
SD: Standard deviation
PVT1: Plasmacytoma variant translocation 1
LINC00667: Long intergenic non-protein coding RNA 667
