# Supplementary figures and images for: LINC00667/miR-449b-5p/YY1 axis promotes cell proliferation and migration in colorectal cancer
Source: Cancer Cell Int. 2020 Jul 17;20:322. doi: 10.1186/s12935-020-01377-7 (PMC7368754; doi:10.1186/s12935-020-01377-7)

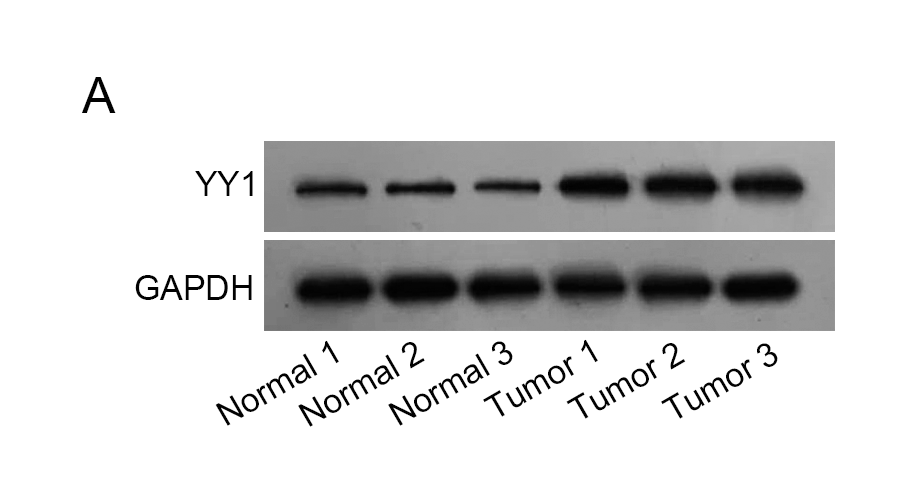

Supplement: Supplementary file 2 — Additional file 2: Figure S1A. The protein level of YY1 in three pairs of CRC tissues and adjacent normal tissues was measured by western blot analysis. N=3 in each group. [file 12935_2020_1377_MOESM2_ESM.tif]
